# Local interaction strategies and capacity for better care in nursing homes: a multiple case study

**DOI:** 10.1186/1472-6963-14-244

**Published:** 2014-06-05

**Authors:** Ruth A Anderson, Mark P Toles, Kirsten Corazzini, Reuben R McDaniel, Cathleen Colón-Emeric

**Affiliations:** 1 Duke University School of Nursing, DUMC 3322, Durham, NC 27710, USA; 2 University of North Carolina at Chapel Hill, School of Nursing, 1200 Carrington Hall #7460, Chapel Hill, NC 27599, USA; 3 McCombs School of Business, The University of Texas at Austin, Texas, USA; 4 Veterans Administration Medical Center, Geriatric Research Education and Clinical Center, Durham, USA; 5 Department of Medicine, Division of Geriatrics, Duke University School of Medicine, Durham, USA

**Keywords:** Nursing homes, Management, Staff interactions, Complexity science, Case study

## Abstract

**Background:**

To describe relationship patterns and management practices in nursing homes (NHs) that facilitate or pose barriers to better outcomes for residents and staff.

**Methods:**

We conducted comparative, multiple-case studies in selected NHs (N = 4). Data were collected over six months from managers and staff (N = 406), using direct observations, interviews, and document reviews. Manifest content analysis was used to identify and explore patterns within and between cases.

**Results:**

Participants described interaction strategies that they explained could either degrade or enhance their capacity to achieve better outcomes for residents; people in all job categories used these ‘local interaction strategies’. We categorized these two sets of local interaction strategies as the ‘common pattern’ and the ‘positive pattern’ and summarize the results in two models of local interaction.

**Conclusions:**

The findings suggest the hypothesis that when staff members in NHs use the set of positive local interaction strategies, they promote inter-connections, information exchange, and diversity of cognitive schema in problem solving that, in turn, create the capacity for delivering better resident care. We propose that these positive local interaction strategies are a critical driver of care quality in NHs. Our hypothesis implies that, while staffing levels and skill mix are important factors for care quality, improvement would be difficult to achieve if staff members are not engaged with each other in these ways.

## Background

Amid persistent evidence of shortcomings in nursing home (NH) care, we expect that management practices influence safety, health outcomes and satisfaction with care [1,2]. Management practices that focus on relationships among staff, such as building connections and developing existing strengths as resources for solving problems, are suggested to be more effective than traditional hierarchical approaches [3]. For example, recent studies describe examples of management practices that influence the capacity of NH staff to work together, including: (a) staff participation in decision-making [4], (b) diversity of thought and actions [5,6], (c) information exchange among staff and managers [7,8], and (d) recognition of effort and rewards for performance [9,10]. Studies have found that when managers in NHs focus on improving relationships there are significant positive patient outcomes such as prevention of decubitus ulcers [11], reduced urinary incontinence [12] fewer medical errors [13], and better uptake of evidence-based practices [5].

However, little is known about how managers develop relationships among staff to improve performance, under what conditions such management approaches are effective, and how they might influence adoption of new knowledge by staff. Therefore, we conducted longitudinal case studies to explore staff and managers’ descriptions of management practices and relationship patterns and we explored their explanations about how they work to achieve better outcomes.

### Guiding framework

**Figure 1:**
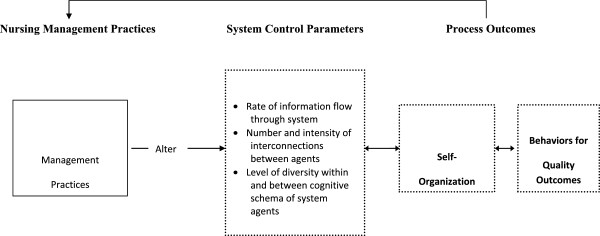
**Conceptual model depicting expected relationships between nursing management practices, system control parameters, and self-organization in nursing homes.** Reprinted with permission of Wolters Kluwer Health: [3].

This study was guided by complexity science. Viewing organizations as complex adaptive systems draws attention to relationship networks through which diverse workers interact with each other to meet their work demands [3,14]. The relationships among workers are nonlinear, which means that the outcomes cannot be directly controlled by managers using traditional hierarchical approaches [15]. In Figure 1, we show how management practices might influence resident outcomes through their impact on staff interactions. As shown, management practices that: 1) encourage diverse staff members to connect in high quality relationships; 2) encourage them to increase the rate of new information exchange about resident care; and 3) encourage them to incorporate diverse perspectives, will likely facilitate staff efforts as they self-organize to meet work demands. Self-organization is the spontaneous emergence of behaviors by the staff; they are the result of the interactions through which members mutually adjust their behaviors in ways they believe will help them meet their goals [16]. Because emergent behaviors are spontaneous, managers cannot directly control them.

Complexity science describes the nonlinear relationships among agents (workers) as the engine through which system improvement emerges as novel and coherent patterns of behavior arise. These nonlinear relationship interactions take place within a structure, such as the NH organizational context with its rules and regulatory environment. This context both enables and constrains emerging properties. Thus, as shown in Figure 1, if managers use approaches that constrain relationship development, new information flow, and incorporation of diverse opinions, self-organization among the workers will still occur but the emergent behaviors will most likely not be effective. Constraining system parameters depicted in Figure 1, and defined in Table 1, is a typical approach in NH management. For example, research suggests that NHs have common barriers to problem-solving such as not including Licensed Practical Nurses (LPNs) and Nurse Aides (NAs) in decision-making [7,17,18], poor communication between provider groups [19], and over-reliance on hierarchical management [9,11,20,21].

**Table 1:** **System parameters**[22]

**Parameter**	**Definition**
Interconnections	Networks of interconnection develop when staff members interact to complete work [23]. Interconnections create social networks and feedback loops through which tacit knowledge develops and grows; local changes in behavior can give rise to system-wide change [24], influencing the extent to which the organization is capable of new behaviors.
Rate of new information flow	Staff members share information with each other about patients and work processes. The rate with which staff in a system share new information will influence generation of new behaviors [22].
Diversity of cognitive schema	Cognitive schemas arise from social, educational, or cultural backgrounds, organizational roles, and age [25]. They are mental models by which people evaluate and make sense of information [26]. Diversity of cognitive schema refers to the variety of mental models available with which to expand possibilities for understanding and acting in novel ways.

Individuals and their immediate work groups are subsystems within a NH. All of these subsystems work both independently as well as mutually and reciprocally. No subsystem is independent of the structure of which it is a part. Structure and process are interconnected, and the relational basis of systems is key to understanding them [27]. These concepts of complexity science drew our attention to staff connections and relationships as well as the ways staff members responded to each other in shaping their work behaviors.

We explored three research questions: (1) What do staff and managers describe as the relationship patterns and management practices in use? (2) What intended, or unintended, consequences do staff and managers associate with relationship patterns and management practices in use? (3) What model best describes and explains the relationship patterns and management practices that foster better outcomes?

## Methods

We used a comparative, multiple-case study design [28] to explore different perspectives of staff members about relationships and nursing management practices [29]. The case method involved in-depth data collection from a variety of sources over six months and resulted in case descriptions and cross-case themes [30]. The methods are described briefly below; find detailed methods in Appendix A.

### Ethics and consent

The protocols of this study were approved by the Duke Medicine Institutional Review Board for Clinical Investigations and underwent annual renewal (Pro00009895). Written permission to conduct the study was obtained from both the administrator and director of nursing in each case study NH; all individuals involved in depth interviews signed informed consent. This study conforms to the qualitative research review guidelines of relevance, appropriateness, and transparency (RATS) [31].

### Sample

Eligible NHs (N = 48) were within 80 miles of Duke University, not in a hospital, and participated in Medicare and Medicaid. To increase the potential for observing variations in relationships and management practices, we purposefully selected NHs that were identified in periodic state regulatory surveys as having higher (N = 2) and lower quality (N = 2).

### Data collection procedures

Each case study was conducted over 26 weeks. Data collectors observed staff members in NHs as they worked including formal meetings, change of shift discussion, and informal encounters between staff members. Staff members from all departments were included (e.g., nursing, medical team, social work, rehabilitation, dietary, environmental services, administration, activities, and maintenance). Descriptive observations laid a foundation for in-depth interviews with key informants. The analysis team developed an understanding of each NH’s context that could be explored using standardized interview questions in the semi-structured interview guide (see Appendix B). Documents and archival records, such as memos, policies, organizational charts, or regulatory reports were obtained to triangulate evidence. Table 2 summarizes the types of participants and data collected in each case.

**Table 2:** Case nursing homes and participant description and data sources

**Facility (Case Number)**	**Case 1**	**Case 2**	**Case 3**	**Case 4**
**Number of staff: total for facility**	104	119	97	86
**Total number of transcripts or documents coded**	142	155	100	119
**Staff participant characteristics**				
% female	84%	81%	78%	74%
Race/Ethnicity				
Caucasian	46%	60%	25%	38%
African American	44%	35%	59%	56%
Other (Asian, Hispanic or unknown)	10%	5%	16%	6%
Age group (years)				
20-24	7%	11%	18%	8%
25-34	22%	32%	27%	20%
35-44	26%	32%	28%	25%
45-54	22%	14%	21%	18%
55+	23%	11%	6%	29%
Tenure in years: mean (SD)				
Tenure in facility	7.6 (9.4)	3.0 (3.0)	1.6 (2.6)	7.9 (9.5)
Tenure in position	6.8 (8.1)	2.6 (3.0)	1.4 (2.4)	6.0 (7.0)
**Facility staff by position involved in observation and/or informal interview**				
Nursing home administrator	1	1	3	1
Director of nursing	1	1	3	1
Assistant director of nursing	0	1	2	0
Quality assurance registered nurse (RN)	1	0	0	0
RN supervisor	2	2	1	1
Licensed practical nurse supervisor	0	0	0	1
MDS nurse (RN)	2	2	2	1
Staff development coordinator (RN)	1	0	0	2
Staff development nurse (RN)	0	2	0	0
Rehabilitation director (or speech therapist)	0	1	2	1
Rehabilitation/Restorative Staff	2	10	3	7
Staff RN	9	6	3	2
Staff LPN	5	12	13	11
Agency LPN	1	0	5	0
Nursing assistants (including Medication techs)	36	36	26	21
Sitter	1	0	0	0
Medical doctor	1	2	2	1
Nurse practitioner	1	1	0	0
Psychiatrist	0	1	0	1
Podiatrist	0	1	0	0
Lab tech (contract)	0	0	0	1
Dietician	0	1	0	0
Dietary manager	1	1	3	1
Dietary staff/aides	10	4	0	4
Activities director	1	1	1	2
Activities assistants	2	1	1	1
Environmental services manager	1	4	1	1
Environmental services staff	8	6	5	5
Social worker	0	0	0	1
Social services staff	2	2	2	0
Administrative support staff	14	13	11	10
Corporate staff	1	7	8	5
*Total # staff*	*104*	*119*	*97*	*82*
**Other observations**				
Shadowed (in-depth observation)	14	26	18	14
Depth interviews	32	39	24	31
Documents reviewed	42	39	20	44

### Analysis

Research team members met weekly to discuss the analysis, refine the codebook, identify emerging themes and write narratives. Every text document was coded by at least two coders using Atlas ti [32].

### Within-case analysis

We used inductive manifest coding of all data [33], followed by meaning condensation [34]. Practicing coding as a team, we developed consistency across coders and identified the appropriate unit of analysis [35] to include large enough chunks of text to keep the meaning in context. When all data were coded, we used data display matrices to triangulate data from multiple sources, participants, and points in time. We then condensed coded data and described themes that depicted the emergent capacity of NH staff members for care [29]. Within-case analyses resulted in four case reports that provided the starting point for cross-case analysis.

### Cross-case analysis

Displays [30] were used to synthesize and compare results across NHs, such as conceptually ordered and case-ordered matrices and causal networks. The cross-case analysis facilitated our identification and description of recurring patterns of management practices and emergent capacity for resident care; it also drew attention to disconfirming evidence and prevented premature or false propositions [28]. The outcomes of cross-case analysis were conceptual models that display new understandings of the links between relationship patterns, management practices, and outcomes.

## Results and discussion

We found that all staff in NHs used both positive and less useful management practices for relating to one another; these were unevenly but widely dispersed in the interactions among staff. We describe the findings first using the umbrella concept described in data which we refer to as “local interaction strategies” (LIS). Then we describe the two patterns of LIS we observed: (a) The Common Pattern of Interaction, and (b) The Positive Pattern of Interaction.

### Local Interaction Strategies

By exploring both relationships and management practices, we found that participants described two different types of management practice. Although standard “management practices” such as staffing and reward systems were discussed, a significant proportion of participants described a second concept that encompassed how people in the NHs interacted with each other, and the resident care outcomes they believed resulted from them. We refer to this second subset of management practices as LIS. A licensed practical nurse explained,

“A lot of people will say, ‘Well, the receptionist just answers the phone.’ She observes a WHOLE LOT. And I’ll ask for her opinion on certain residents and how they’re acting… Housekeeping [will tell you] any complaints a patient may have…the food, the service, that kind of stuff…because the resident can pick anybody to talk to… [and] might tell them something that they would not tell anybody else.”

We noted several LIS in the licensed practical nurse (LPN’s) comment: (a) she formed relationships with staff of all types, (b) she used her relationships as resources to gather information to guide care, and (c) she focused her attention on what was best for the residents. Her interactions with other staff members in her immediate or “local” environment improved the capacity for providing patient care. As she connected with others, she became aware of their expertise and opinions; as she sought information and listened to others, she discovered new information important for guiding care; and, as she paid attention to feedback of others and integrated multiple points of view, she learned to make sense of patient needs and challenges around her. We found that these LIS were dispersed in the behaviors of staff members within and across the case study NHs, irrespective of staff members’ demographics, role, or tenure in the NH. Thus, this subset of management practices was much more than a set of strategies that managers used to coordinate or organize the work of their teams; it was a set of behaviors that staff members of all types used while interacting with others to negotiate the social context of their work.

We observed three qualities of staff interactions that were consistently associated with a greater capacity of NH staff members for providing care: they were transient, they occurred in the immediate or “local” context of staff members at work, and they involved the behavior of one or more staff members that made it easier or possible to achieve the objectives of work. For example, we observed that staff members who “pitched-in to help others” consistently increased the capacity for effective care. A certified nurse assistant (CNA) explained, “Staff members [here] are so caring; you can really get help, like [Nurse Supervisor], she will put her heart into helping. She does not mind getting her hands dirty.” In the CNA’s comment, we noted that the nurse supervisor’s willingness to pitch-in resulted in the CNA’s feeling that the people she worked with were “caring,” expanding their capacity for working together. As we observed the nurse supervisor and others like her, we discovered that they strategically interacted with others in ways that maximized the potential for getting work done, simultaneously providing good care for residents and creating a supportive work environment.

We identified a variety of LIS, some of which participants linked to mediocre to poor outcomes and others they linked to good to excellent outcomes. Their descriptions suggested that use of LIS occurred in patterns and these patterns were transient. For example, when certain patterns of LIS were used, staff responded with energy and enthusiasm that resulted in better system outcomes. When other LIS patterns were used, however, staff responded with exhaustion and withdrawal of effort from which worse system outcomes emerged. We observed these two patterns in all NHs, irrespective of the NH’s overall quality ratings. We did not find managers or staff members who consistently used LIS of one type or another. Most often, the participants described LIS that limited engagement with others or engendered feelings of disrespect; this pattern we labeled the “Common Pattern.” Less frequently participants described LIS that facilitated robust connections, information exchange, and diversity in cognitive schema: this pattern we labeled the “Positive Pattern.” Staff explained that the work environment and resident care practices that emerged from these patterns were either effective and satisfying or negative.

### The common pattern of local interaction

**Figure 2:**
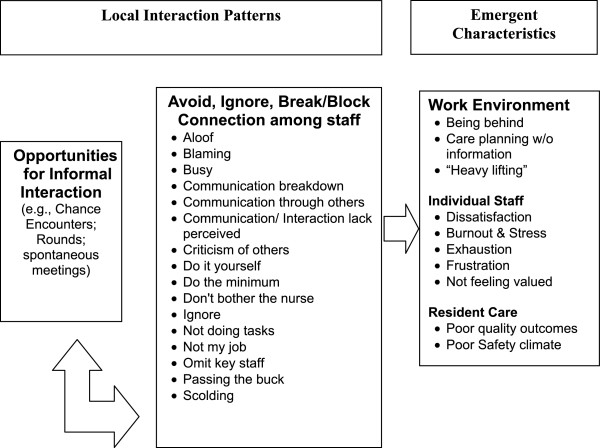
**Common interaction patterns and emergent system characteristics.** When staff and managers have opportunities to engage each other, either formally or informally, the types of interactions that occur are important to the outcomes that emerge. When interactions encourage avoidance of each other and issues, the emergent outcomes for the work environment, staff, and residents are poor.

The default interaction behavior for many managers and staff members fit the Common Pattern (Figure 2), in which people blamed others, avoided collaboration, decided ‘not to bother the nurse’ , ignored others, said ‘it’s not my job’ , scolded others, or ‘passed the buck’. As staff members avoided interaction, their diminished information and ability to self-organize effectively increased the risk for harm to residents, in even the most commonplace situations. A nursing aide’s explanation of her avoidance of licensed nurses epitomizes the diminished capacity associated with the Common Pattern. The nursing aide stated,

…they’re not that much help…Say you’re in the room with a resident who’s an accident waiting to happen. You don’t feel comfortable leaving him… And then, there is another lady who’s accident-prone…and her alarm goes off. Instead of the nurse answering it, they just sit there listening to it and then they’ll come [tell us] to take them to the bathroom. That’s why I don’t even bother now [to ask the nurses for help]… You get in the habit of…getting another nurse aide.As displayed in the Common Pattern (Figure 2), the nursing aide’s decision to ask only other nursing aides for help was likely a consequence of her prior interactions with nurses. This nursing aide had learned that nurses on her unit would not pitch-in to help; the nurses’ prior interactions with the nursing aide did not contribute to or reinforce connections with the nursing aide. Thus, when she really needed help to keep residents safe, the strength of a unified nursing team was not available and the consequences were twofold: a greater risk for resident harm and the nursing aide’s feeling of being unrecognized by her charge nurse.

The relationships between NH managers and staff members often conformed to the Common Pattern; in particular, managers missed opportunities to listen and notice staff contributions. For example, when mid-level managers conducted audits to ensure that work was completed according to policy, they frequently punished staff members when they might have listened and gathered information to help staff members develop greater skill. Staff members explained that the managers paid attention to a narrow range of their work, such as whether socks were stored in the drawers and the linen carts were covered, rather than thanking the staff or noticing the good things done in challenging circumstances. For example, we observed a supervisor who found a bottle of shampoo in a clean linen cart and scolded the nurse aide for this infraction; we later learned how the manager’s behavior affected the nurse aide. The nurse aide told us she bought the shampoo with her own money because she believed that a resident did not respond well to the facility’s standard shampoo, and was frustrated that she was punished for her extra care. Although the supervisor’s concern may have been justified (e.g., ensuring the hygiene of linen cart), the punitive interaction with the nurse aide damaged an important relationship and prevented the supervisor from learning details about a resident’s care, and discouraged the nurse aide from “going above and beyond” for residents again.

Interactions described in the Common Pattern not only limited interconnections and information exchange among staff, they also created formidable barriers to problem solving. The comments of one nursing aide highlight the most prevalent gap in problem solving in the NHs—limited cognitive diversity (e.g., participation by staff of all types) in solving routine, “front-line” problems of resident care.

CNA: If I could change something around here I would start with the front office…How are we going to work together when they don’t?

Interviewer: So do you get much contact with anyone in the front office?

CNA: When they get ready to come out here and chew somebody out about something, I see them then.

Interviewer: …Do you have meetings?

CNA: No…But that is what it boils down to, no communication. …we come in and work blind. That is how it is here.

In our analysis of the case data, the CNA’s statement, “…we come in and work blind,” was a striking example of how staff interactions in the Common Pattern reduced the capacity for solving resident care problems together. We observed clinical problems, processes of care, meetings for exchanging information, and routines for custodial care that succeeded or failed based on the way staff members gave or received feedback, searched for alternative solutions to problems, and tried to make sense of problems. Consistently, we observed that staff interactions which created barriers to seeing problems from multiple viewpoints and debating alternatives limited the capacity for the care that residents needed.

Another aspect of interactions that fit the Common Pattern was strong reliance on rules and policies while ignoring what was evolving in the current environment. This created a lack of openness for new ideas or frontline staff input in decision-making. For example an administrator stated, “But as far as decision-making for [floor staff] on a daily basis, I don’t really think there’s a lot of that, simply because the systems are in place that they know they need to follow and it’s pretty standard.” In the same facility the interviewer asked, “Can you give me an example of something that might be out of the ordinary? The assistant director of nursing stated, “[Sighs] [Pause] Hmm. [Pause] Not really, because just about everything is covered [in the rules].

In summary, interactions in the Common Pattern did not support good relationships among staff, the flow of information, or inclusion of a variety of perspectives in decision-making. Instead, these patterns tended to reduce staff members’ willingness to report their concerns about residents and to increase staff members’ feelings that they were working without sufficient information about residents in their care. Staff also described job frustration and fatigue because they were not able to do their best for residents.

### The positive pattern of local interaction

**Figure 3:**
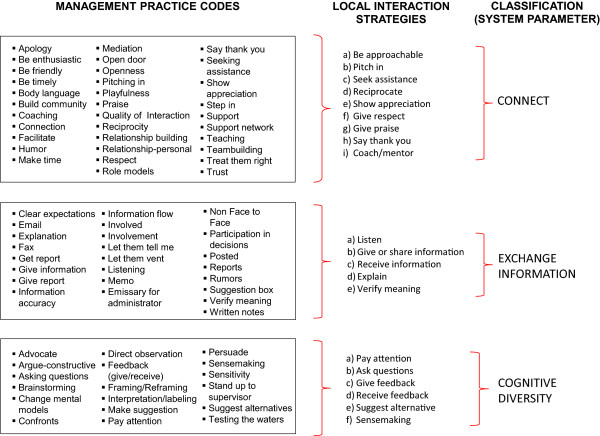
**Local interaction strategy codes and themes.** Coding the case study text, we identified over 80 management-practice codes which were then condensed into 20 local interaction strategies that were further condensed using Stacey’s [22] system control parameters, into three themes, e.g., connection, new information exchange, and cognitive diversity.

Interaction strategies that fit into the Positive Pattern were derived from a set of over 80 codes in the case data. These included interaction strategies such as “be enthusiastic,” “praise,” “give information,” “let them vent,” “brainstorming,” and “make suggestions” (Figure 3). We condensed these into 20 “positive local interaction strategies.” We organized these 20 LIS into (a) interactions that promoted new *information exchange* (i.e., listen, give or receive information, explain, or verify meaning); (b) interactions that promoted *staff interconnections* (i.e., be approachable, pitch-in, seek assistance, reciprocate, mediate, model behavior, or coach); and (c) interactions that promoted *cognitive diversity* (i.e., pay attention, ask questions, give/receive feedback, suggest alternatives, sensemaking). The labels for these highest order themes were derived from Stacey’s [22] three system parameters (see Table 3).

**Table 3:** Local interaction strategies and exemplar quotations

**Local interaction Strategy/ Definition**	**Exemplar quotation**	**Outcome described**
**Connection**: Local interactions strategies that help individuals make and keep good relationships with co-workers and foster good teamwork; these lay the foundation for effective information exchange and problem solving about resident problems.		
• Be approachable--Be open, listen, and respond to what people say	A rehabilitation therapist said, “If I have a positive relationship before a situation comes up, then I’m more likely to get a positive response from [the nursing assistants] out on the floor, too… They will stop me and they’ll tell me about issues.	Respect
		Better care planning
		Early detection of problems
• Pitch-in-- Go beyond regular duties to help others	I [nursing assistant] had one total care person and the rest just need some assistance which made my assignment easier. So I went down and gave a couple of showers and made a couple of beds for the other girls…They did not ask me, I just went ahead and did it.”	Teamwork
		Staff well being
• Seek assistance-- Request help	An employee performance standard (employee handbook), “They are willing to ask for help and do not think they can handle every situation on their own. If they can’t get the needed result they will find someone else who can. They are primarily concerned about getting the right result for a resident.”	Patient safety/earlier detection of problems
• Reciprocate-- Give and take with others in a way that generates goodwill	A business manager says, “I give [my assistant] a lot of my work to help me out – AND we work together. She does things for me. I just don’t like telling her what to do. I said, ‘These are the things that have to be done, let’s work on it together.’ And I do that kind of thing. I think its being a team player and not just unloading everything on one person. … She had to leave early one day and I said, ‘Well, go, I’ll finish up.’ And I made her leave. You know, I believe you should be able to take the load over.”	Teamwork
		Staff well being
• Show appreciation-- Express a positive opinion of other peoples’ actions	“If I [administrator] see things that are right…we write you up for that in a positive way…we try to focus on what you are doing right.”	Motivating
		Empowering
		Staff well being
		Confidence
		Satisfaction
• Give respect--Let others know you value them and their opinion	An administrator says that “to develop employees to their fullest potential and allow them to see the difference that THEY can make in people’s lives….by viewing every [resident] as an individual, whether [or not] they are capable of communicating with us to treat them with the dignity and respect that THEY deserve and to – to try to give them the best quality of life. I feel like if we treat our employees right, they’ll treat the residents right. But, we have to model the behavior that we want them to have. And, uh, so – and I think that starts with - with EVERYONE, but certainly with me. And so I do everything I can to encourage that behavior.”	Respect
		Staff well being
		Patient quality of life
• Say thank you-- Express gratitude, pleasure and satisfaction	“Staff like to be recognized…when [the administrator] goes in there one-on-one and says, ‘thank you for the hard work that you do’ and ‘can’t even pay you enough for the hard work that you do. We really appreciate it.’ The staff like that.”	Staff well being
		Respect
• Give praise-- Let others know you admire the work they do	“It makes me [nurse] feel good when I do something good for a resident, like if something happens to them and I end up having to send them out…and someone will come to me and say, ‘You did a really good job. You knew to call 911, you knew to do this. You knew to do that.’”	Respect
		Motivating Empowering
		Staff well being
		Satisfaction
• Coach/Mentor-- Guide, instruct, or train others; form trusting relationships	“I [nurse] use to get the worse nursing assistants. But what I would do was to…work with their strengths and whatever weaknesses. I would teach them. ‘You don't leave up a rail’…I will go back and check behind them. Before long they will pick up on these things”	Valuing Confidence
		Better resident care
		Patient safety
**New Information Exchange**: Local Interaction Strategies that help individuals give and get new information about residents to and from the right people, in a way that ensures everyone understands.		
• Listen-- Hear with thoughtful attention	A nurse manager said, “Just let people know that they’re *important* [said with emphasis] and that what they have to say is important… Whether you can do anything or not, at least you’re listening! You know that you’ve heard what they’ve had to say.”	Staff feel respected
		Valuing
• Give information-- Share information; give a report	After a CNA described how she solved a resident behavior problem, a researcher asked, “Did you share your strategy with anybody else?” The CNA said, “I did with another nursing assistant. She had [the resident] the next day and I told her what I did… she said okay and she tried the same thing and it worked.”	Teamwork
		More consistency in resident care
• Receive information-- Graciously accept information	An LPN explained, “If you’ve got concerns or complaints, being able to go to someone and say, you know, ‘This is bothering me,’ or ‘I’m concerned about this.’ And, them at least addressing your concerns—that’s important. They may not do anything about it THEN, but then at least they LISTEN to you. I think that important.”	Potentially pick up resident issue
		Staff feels respected
		Staff well being
		Valuing
• Explain-- Give more details to clarify what you mean	A housekeeping director said, “If you just say, ‘This is wrong you know, you better change this, it is not good,’ they [housekeeping staff] will just end up hating you…I explain to them, ‘This is what you need to do. Bring the trash can; put a lining on that trash can, so when you take off the diaper, drop it directly in…I give them several examples like that and that way they understand.’”	Staff well-being and efficacy
		Better job performance
• Verify Meaning-- Make sure you understand information shared by others.	A nursing home administrator relayed a conversation with an employee who said, “I don’t understand why I’m submitting a doctor’s bill.” I said, “Prospective Payment System…” and I went through the scenario. She said “Now I understand.” That’s what I try to … I’ll look in your eyes; if I’m comfortable you got it, we move on with business. If not, I want you to think about it for a minute…	Delegated tasks more likely to be done properly
**Cognitive Diversity**: Strategies that help individuals notice changes in residents’ conditions and that they act on what they see and discuss multiple opinions to make good sense of the problem and ensure shared meaning of events.		
• Pay attention-- Make a conscious effort to stop, watch and act.	“In the wheelchair…in front of the nursing station, one male resident had been snoring very loudly. A certified nursing assistant moved him and another resident in a geri-chair around and reclined both so they can sleep more comfortably. She then picked up a couple of paper towels that had fallen on the floor and went into a bathroom to throw them out and wash her hands.”	Better quality
		Patient safety
		Earlier detection
• Ask questions-- Ask for explanation when you feel uneasy about something and when you feel you were not heard.	A nursing assistant said, “I would…look at the chart. But…for me to really, really understand the resident - since that nurse knows that resident, I would rather go to [the nurse] and say, ‘Well, how about so and so, what kind of person is he?’ You know, ‘can he stand, can he sit?’	Patient safety
		Better care
• Give feedback-- Provide others with useful opinions or reactions to their work	A physical therapist said, “If I hear of problems, I try to pull people in. For instance, we’re having issues with some restraint situations…And so, we’ve opened up some lines of communication.”	Patient safety
		Better care
• Receive feedback-- Graciously accept others’ opinions about, or responses to, your work	A Minimum Dataset Nurse explained, “I have a particular resident who…filled my ears full yesterday and, you know, I explained to him that I’ll be coming to him every day that I’m here - sitting down and talking with him and, you know, I’m going to help him, you know, try to resolve anything that I can. And he was very responsive to it.”	Quality improvement
		Resident quality of life
• Suggest alternatives-- Give different options for others to consider before taking action	A Nurse stated, “I looked in on a patient, but, she’s been in the bed for the last few days…So, I asked [the charge nurse], ‘Why is she in the bed?’ And they said, ‘Oh she just hasn’t been feeling well.’ So, I tell the charge nurse, ‘You might want to look for a UTI, because she has a history of having UTIs.”	Earlier detection of problems
		Better care outcomes.
• Sensemaking-- Talk with other people to ask, “what does this mean?” Together make sense of confusing information or situations	An Activities Director said, “There was an issue with [a resident] trying to sneak food. And he, at the time, couldn’t take anything by mouth. We had a lot of conversations…everyone could come with their ideas and really try to think about how to make it work for the resident and the family…It was great. It was a lot of conversation and really coming together for the resident’s best interest.”	Better care planning and decision-making
		Better care outcomes

We observed the “Positive Pattern” less frequently, but it emerged when either managers or staff members purposefully used the positive LIS for building capacity to provide good care and create satisfying work environments. Again, these positive LIS were observed in behaviors of people in *all* job categories, not merely managers, suggesting that optimizing capacity requires that both managers *and* staff manage relationships. Staff explanations linked these positive LIS behaviors to the NH’s ability to provide better care. We did not find any single NH in which all people used the LIS in the Positive Pattern, nor did we find any single individual who used the Positive Pattern consistently. However, we saw transient examples of positive LIS working well and at different times and locations in every facility, suggesting that most NHs have some existing capacity to employ this positive pattern.

**Figure 4:**
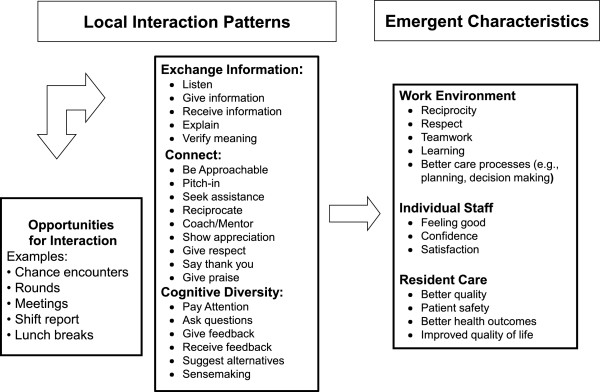
**Positive pattern: model of local interaction strategies and emergent system characteristics.** When staff and managers have opportunities to engage each other, either formally or informally, the types of interactions that occur are important to the outcomes that emerge. When local interaction strategies facilitate high quality connections among staff, exchange of new information, and use of cognitive diversity, the emergent outcomes for the work environment, staff, and residents are positive.

As depicted in Figure 4, we observed that positive LIS fostered connections between staff members, allowing for new information exchange and diversity of cognitive schema. Staff described that purposive use of positive LIS helped them to provide better care and create better work environments. Connection strategies, such as pitching-in, being approachable, and coaching others (Table 3), are defined as those that help individuals make and keep good relationships with co-workers; these lay the foundation for information exchange and problem solving about resident problems. Several of these strategies are illustrated in a nursing aide’s description of how she worked with a peer to meet a care challenge.

“Today I was very disturbed. [Resident name] had diarrhea… I tried everything. I even let her get it all out, thought she was finished, came back, dried her up again, come back, she did it again. I said, “Oh, God, am I ever going to get out of this room… I was *mad* because it was slowing me down. I had four more people that needed to be up.”

The nursing aide continued,

“I went and got [name of another CNA], and let her handle it. I asked her to help me out. So, she took over!….Every day we have a routine. We work together. …In the morning we’re on our own unless we need help, but in the afternoons, we do our rounds together. She’s there, helps me catch up. She’s my buddy…It gets a whole lot of work done faster.”

When the interviewer asked, “do you do that for her too?” the CNA responded,

“Uh huh, all the time…it’s all about teamwork…it works excellent too. [In the past] you go home tired and worn out - don’t have any energy left to play with your kids… [Now] when you go home, you’re not all drained out and tired.”

This CNA’s story described positive work outcomes for nurse aides who used these interaction strategies frequently enough to establish positive patterns; *pitching-in* and *being approachable* were especially powerful interactions that were described to have positive outcomes (e.g., trust, feelings of control and competence, and support) and better care for residents.

These connection strategies in the Positive Pattern frequently created a cascade effect, generating opportunities for *information exchange*. As defined in Table 3, LIS that promote information exchange ensure that all necessary staff members give, receive and understand information about residents in a timely manner. We observed that staff members who carefully listened to what others were saying created pockets of excellence in care. A physical therapist explained,

“[I tried] to be very patient whenever I tried to get [nursing] to do [a new task/skill]… Oftentimes, they would not follow through and I tried not to lose my temper or yell, because I know everybody has a learning curve…I try to interact with nursing staff in a way that facilitates good dialogue [so] they feel that they can *approach* me with patient problems, so that we can solve things together. I listen to what they have to say, and their way of doing things, and try to see if that’s the best way to meet the patients’ needs.”

In this positive interaction pattern, the physical therapist did more than extend courtesy to her nursing colleagues; she listened, she was open to new ideas about “the best way to meet the patients’ needs.” The therapist’s openness made communication easier and helped nurses to recognize her as a resource for solving problems in care.

In another example, staff used a positive LIS we labeled “coach/mentor”, defined as creating opportunities to help others develop their ability to care for residents. A nurse explained, “I used to get the worst nursing assistants…[however,] I work with their strengths and whatever weaknesses. I would teach them… Before long they will pick up on these things.” As the nurse engaged with the strengths and weaknesses of the new nursing aides, she was able to help the nursing aides learn, thereby increasing the capacity of her team for better resident care and patient safety.

Staff members described *connections* and *information exchange* as the necessary foundation to engage in a particularly powerful set of positive interaction strategies, those which promoted their ability to use *cognitive diversity.* We defined these as strategies that help individuals notice potential for harm and work in groups to consider multiple opinions, make sense of the problem, and ensure coordinated actions (Table 3). An important first step toward cognitive diversity was when staff at all levels engaged in *paying attention*.

“ADON: I don’t get upset very often - but when a patient passed away - one of the family members had come down the hall … and overheard a CNA say, ‘Yeah, you know, when, a family member’s dying, the family comes out of the walls like vultures.’ [In a tone of incredulity] I said, ‘How could anybody do that?’ I said, ‘Put yourself in their place. How would you feel if your mother had just died and you heard somebody say that?’”

Although her tone may have conveyed annoyance, we see in the DON’s interaction with the CNAs that she tried to use this as a learning experience. Instead of reprimanding the CNAs, she helped them see the situation with empathy. She did this by *paying attention* and acting on what she observed, in this case the family’s complaint. She then *asked questions* to help the CNAs explore the situation from multiple points of view. This interaction helped the CNAs to make *different sense* of the experience, which might help them behave differently in the future. A staff development nurse elaborated on these types of interaction strategies, explaining that managers and nurses must first change the way staff think about situations in NH care before they would be able to learn the desired behaviors and approaches to resident care. This is a form of what we have termed *sensemaking*. In this illustrative example, the staff development director helps staff make new sense of their behavior:

“Staff Development Director: If I see something wrong, …I explain to them why. Because if you just say, ‘This is wrong, you better change this’ , there is no good effect. They will just end up hating you. But, if you say, for example, ‘You know that the soiled diaper that you lay down on the floor is dirty and contains different microorganisms that …will make you all sick…I explain to them and so they learn…that way they understand and they comply.’”

Finally, listening to other perspectives and working together to make sense of a situation (*sensemaking*) was described as a way to foster more effective actions for residents. A nurse manager stated, “Some people began to have an appreciation for that and could do that—really talking to people, looking at things from other peoples’ perspective. And then [we decided] …if you can’t do these things, then this isn’t the place for you.” A rehabilitation director explained:

“If I hear of problems, I try to pull people in. For instance …the way restraints are handled…is very heavy on [physical therapist]. She’s really carried the burden for YEARS… And so, we’ve opened up communication …. Pulling us all into the same room to be able to hash out what needs to be done [going] forward. It’s a multi-disciplinary approach versus a physical therapy approach - It shouldn’t fall to one person; what if she’s not here? Having a multi-disciplinary approach is the biggest key. It’s not just one person LEADING THE CHARGE….[It] means change. Change is often hard.”

In summary, staff interactions that promote cognitive diversity contribute to resident safety, the quality of care and changing staff behaviors. Staff descriptions of these interactions also suggest that cognitive diversity can only be achieved when staff members first use interaction strategies to build connections and exchange information. Thus, the set of positive LIS must be used in combination to be most effective.

### Discussion

Theories of complex adaptive systems (CAS) suggest that patient and health system outcomes emerge from the nonlinear interactions among staff. Emergence is the arising of novel and coherent structures, patterns and system properties [27]. Our case study participants described patterns of interactions; they also explained what they believed to be system outcomes in terms of care quality. In adaptive systems, the nature of what emerges is uncertain but might be estimated to fall within a range of outcomes such as poor to mediocre or good to excellent [36]. Our case study data revealed two patterns of interactions that lead to qualitatively different outcomes. The Common Pattern of interaction (Figure 2) was linked to care outcomes described as mediocre to poor and the Positive Pattern of interactions (Figure 4) was linked to care outcomes described as good to excellent. Both patterns were described in every NH, regardless of the measure of overall NH quality, and this supports Rosen’s assertion that in a CAS, causal patterns are intertwined and no one pattern will describe a system [37]. Also, CAS theory suggests that the emergence of outcomes can be fueled by interactions originating from the top-down or bottom-up [27], which supports our finding that staff members at all levels of the hierarchy used both patterns of interaction. This finding suggests the need for a whole system approach when trying to improve interactions in the NH, both top-down (e.g., managerial interventions to cultivate the Positive Pattern) and bottom-up (e.g., organizational interventions to cultivate staff relationships and new patterns of interaction). Finally, CAS theory states that these types of nonlinear interactions take place within and are not separate from the structure of the system in which they occur, and that these structures both enable and constrain emerging properties. Therefore, local interactions in NHs are critical to system outcomes, but it must be remembered that they take place within the organizational structure, such as a strong top-down managerial approach, and these structures in turn influence the nature of interactions and outcomes. The implication is that structures are necessary but not sufficient to understanding system dynamics and outcomes that emerge.

Even though we only intermittently observed the positive pattern of relationships, they created evidence to hypothesize that if staff would use the set of positive interaction strategies consistently, they would have the ability to increase the capacity of NHs to improve care with existing staff. The fact that we saw these behaviors at least intermittently in every NH suggests that existing capacity is present in NHs and it that can be more widely developed. Rantz et al. [38] came to a similar conclusion in their quality improvement intervention which improved outcomes such as pressure ulcers and weight loss without adding staffing or costs. Our findings are supported by other prior work suggesting that improving organizational capacity has to do with seemingly “ordinary activities” [39], p. 384 that are embedded in social structures that “make use of real-time information, simultaneously explore multiple alternatives, [and] rely on quickly created new knowledge….” [40], p. 919, to facilitate the ability of staff to continuously create solutions to emerging problems.

Unfortunately, this set of positive LIS was not widespread or in regular use in our study NHs. We observed that care tended to be coordinated using centralized and hierarchical approaches which are likely to constrain important social interactions and may limit collective problem-solving [1] (Figure 2). The less common, but positive interaction pattern (Figure 4), led to pockets of excellence in which staff members interacted in ways that coordinated responses to emerging problems in care. This finding suggests that management practices that activate positive local interactions among staff in NHs are critical resources for achieving better care outcomes. Thus, while formal hierarchical structures, policies and procedures are necessary, they are not sufficient for high quality care.

Practice implications arise from the observation that the power of positive LIS was most apparent when they were used (or missing) on the front line. Thus, managers should foster links between frontline nurses and nursing aides and other clinical and support staff. This is likely the most powerful area for LIS to improve care so that observations at the bedside can flow to clinical decision makers and back to the frontline staff, for example through care plans. Managers may consider adding new job performance measures to support and provide feedback for front line staff as they practice new LIS, reinforcing their efforts with praise and acknowledgement. Managers should scan their facility for existing pockets of excellence, to discover, support and expand staff interactions and relationships that already promote better performance. Emerging research [39] suggests that identifying pockets of excellence in NHs may enable managers to isolate relational practices that they can foster and replicate for staff members in other departments or positions.

### Limitations

The case study design requires thick description [41] thereby limiting sample size and geographic diversity. Thus, we do not know if people in facilities outside of this region would offer the same explanations about the influence of LIS on the capacity for high quality care in NHs. Although we purposefully selected NHs with either higher or lower quality indicator scores, these point-in-time measures did not capture longitudinally emergent management behaviors. Therefore, rather than using these aggregate, cross-sectional institutional indicators of quality, we relied on staff descriptions of individual episodes of care and their outcomes. This turned out to be the key to the discoveries made in this study because it allowed us to see pockets of excellence where they occurred in both “high” and “low” performing NHs. Using extended time in the settings and data collected with multiple methods and from multiple study participants [28], we were able to compare findings from diverse points of view and use analysis strategies such as triangulation [42] to identify patterns that indicated higher quality interactions and outcomes. We would have missed the “pockets of excellence” that occurred in lower quality facilities if another method had been used. Knowing that lower quality facilities also have these pockets of excellence, explained by LIS, increases our confidence in our suggestion that LIS can help *all* NHs improve.

## Conclusion

We found that positive local interactions among staff members enabled the emergence of pockets of excellence in NHs. Moreover, we identified specific local interaction strategies that are potential levers to optimize the capacity of staff to improve resident care and work environments. These findings indicate the need for research to test interventions that (a) explicitly foster staff relationships and promote the use of the LIS to improve the capacity for providing better care, and (b) describe the influence of the LIS on staff and resident outcomes.

### Appendix A

#### Detailed methods

We used comparative, multiple-case study design [28] to examine multiple cases to explore different perspectives of staff members about relationships and nursing management practices [29]. The case method involved in-depth data collection over six months from a variety of sources and resulted in case descriptions and cross-case themes [30]. We addressed rigor with strategies for assuming confirmability, dependability, credibility, and transferability [43,44]. We addressed confirmability by using (a) multiple data collectors and data analyzers who served as a check and balance for each other, and (b) consultants with the role of uncovering assumptions and biases of the researchers and assuring that rival hypotheses or conclusions are considered. We addressed dependability by developing clear protocols to assure data collectors used comparable procedures; creating and refining a code book for consistency across data analyzers; and employing weekly coding checks to assess level of agreement and all disagreements were resolved through discussion and the code book updated. We also established an electronic audit trail with details of coding decisions and data reductions. We addressed credibility by explicitly searching for disconfirming evidence; using triangulation of data from interviews with informants from different groups (i.e., administrators, directors of nursing, RNs, LVNs, CNAs) as well as from direct observations and relevant documents and records; and conducting within-case and cross-case analyses to prevent premature conclusions. We addressed transferability by using explicit criteria for the case selection that provided for comparisons with other samples and making rich descriptions of the data to allow judgments about potential transferability.

##### Sample

Eligible NHs (N = 48) were within 80 miles of Duke University, not in a hospital, and participated in Medicare and Medicaid. To increase the potential for observing variations in management practices, we purposefully selected NHs that were identified in periodic state regulatory surveys as having higher (N = 2) and lower quality (N = 2). We calculated composite quality scores from Minimum Data Set Quality Indicators, using the algorithm suggested by Anderson et al. [45]. Two NHs were randomly selected from those with composite scores falling at the 20th percentile or below and the 80th percentile or above. Signed permissions to conduct the study were obtained from the administrator and director of nursing; individuals involved in depth interviews signed informed consent.

##### Data collection procedures

Each case study was conducted over 26 weeks. Primary data collectors (1 MSW and 1 PhD) received one-month of intensive training before beginning fieldwork. Focusing on day and evening shifts, data collectors observed staff members in NHs as they worked and observed formal meetings, change of shift discussion, and during informal encounters with each other. Staff members from all departments were included (e.g., nursing, medical team, social work, rehabilitation, dietary, environmental services, administration, activities, maintenance). Data collectors jotted field notes by hand and typed them daily to ensure best recall. Descriptive observations laid a foundation for in-depth interviews with key informants. They made numerous general observations of work routines and also shadowed staff members (N = 86). During in-depth semi-structured interviews (N = 126) lasting about 45 minutes each, participants described their relationships and experience of management practices in detail, and explained how it impacted the quality of their care. A sample question is, “In what ways do other people here help you do your job better?” (See guide, Appendix B). Interviews were tape-recorded, transcribed and reviewed for accuracy. Documents and archival records (N = 145), such as memos, policies, organizational charts, or regulatory survey reports were obtained to augment evidence.

##### Analysis

The full research team participated in analyzing data using manifest content analysis and thematic condensation. Research team members met weekly to discuss analysis and refine the codebook, identify emerging themes and write narratives. Every text document was coded by at least two coders using Atlas ti [32].

##### Within-case analysis

We used inductive manifest coding of all data [33], followed by meaning condensation [34]. All coders were fully immersed in each case; each participated in some aspect of data collection, spent time in each facility and attended weekly team meetings at which case studies were discussed. All coders read all data and participated in a retreat at the close of each case at which we synthesized the case summary and prepared the exit presentation for the site. To ensure common understanding, the full team discussed any new codes weekly. When a code was added or changed, the analysis team recoded material already examined. Practicing coding as a team, we developed consistency across coders and identified the appropriate unit of analysis [35] to include large enough chunks of text to keep the meaning in context. Coding units were sorted into categories and subcategories that were expanded until all meaningful coding units were captured.

When all data were coded, we used data display matrices to triangulate data from multiple sources, participants and points in time and to condense coded data and describe themes of management practices and the emergent capacity of NH staff members for care [29]. Descriptive and explanatory diagrams of links between management practices and outcomes were developed. Within-case analyses resulted in four case reports that provided the starting point for cross-case analysis. At the conclusions of the four case studies, no new codes or themes emerged as we reached data saturation [29].

##### Cross-case analysis

Displays [30] were used to synthesize and compare results across NHs, such as conceptually ordered and case-ordered matrices and causal networks. The cross-case analysis facilitated our description of recurring patterns of management practices and emergent capacity for resident care; it also drew attention to disconfirming evidence and prevented premature or false propositions [28]. The outcomes of cross-case analysis were hypotheses about links between relationship patterns, management practices, and outcomes, which we summarized in conceptual models.

### Appendix B

#### CNA/NURSE Interview Guide (used with CNAs, LPNs, RNs)

General topic 1 (Question to gather descriptions and explanations about the individual’s personal-level/perspective on Quality of Care:

1. What is it like to be a CNA/Nurse in this nursing home?

2. What things about your work do you do well?

3. What you see as most important in your role?

4. What does good resident care mean to you?

5. Tell me about a time when you were happy because you had done such a good job caring for your resident?

a. What helped you be able to do a good job?

6. Tell me about a time that you were not happy with how things went or you did not feel you did a good job caring for your resident?

a. What things got in the way of doing a good job?

7. What do you see as most important in your role?

General Topic 2 (Question to gather descriptions and explanations about the individual’s descriptions and explanations about the quality of care in this nursing home)

1. What is the quality of care like in this nursing home?

2. Describe what you think is done really well here?

3. What gets in the way of good quality?

4. Use if relevant… There has been a lot of change (turnover) in this nursing home administration, e.g., director of nursing (DON) or nursing home administrator (NHA). What has that been like for you? How do you go about doing your job with all the changes?

a. How do you go about getting to know new staff coming in?

5. In what ways to other people here help you to do your job better? (ask about NHA, DON, Nurse, Nursing supervisor, Minimum data set Nurse, CNAs) (ask about getting information, doing care tasks, etc)

6. What is teamwork like in this nursing home?

7. Who asks you for information about your residents?

8. Sometimes you may have information about your resident that you think others need to know (e.g., CNA, nurse, DON, MD). How do you go about sharing info about your residents with others if they haven’t asked?

9. How do decisions get made here about resident care, schedules, etc.

a. Probe for formal (e.g., care planning meetings, QI meetings) involvement and informal involvement

b. (e.g, nurse/NHA comes to me to ask my opinion, nurse/NHA asks me about things if they happen to run into me in the hall)

10. What staff member do you wish you had more contact with?

a. How would that help you in your job?

11. Ask nurses to talk about their usual daily interactions with CNAs; also

a. explore for relationship patterns with DON, mid-level nurse managers, and medical staff.

General probes for elicit explanations:

a. What got in the way of that working well?

b. What happened? What was meant to happen?

c. What things made it better?

d. Who made a difference and why?

## Abbreviations

CNA: Certified nurse assistant; LPN: Licensed practice nurse; LIS: Local interaction strategies; NH: Nursing home; RN: Registered nurse.

## Competing interests

The authors declare that they have no competing interests.

## Authors’ contributions

RAA was the PI on the study and was responsible for the design and oversight of study execution and analysis. MT participated in analysis and wrote portions of the manuscript. KC and CCE were co-investigators on the study and participated in data collection, analysis, and read and revised drafts of the manuscript. RM participated in designing the study, reviewing analyses, and he read and revised drafts of the manuscript. All authors read and approved the final manuscript.

## Authors’ information

**Ruth A. Anderson**, RN, PhD, FAAN is the Virginia Stone Professor of Nursing at the Duke University School of Nursing, Director of the Adapt Center for Cognitive/Affective Symptom Science, and Senior Fellow in the Center for the Study of Aging and Human Development.

**Mark Toles,** RN, PhD, is a John A. Hartford Building Academic Geriatric Nursing Claire M. Fagin Fellow and is Assistant Professor at The University of North Carolina at Chapel Hill School of Nursing.

**Kirsten Corazzini,** PhD, is Associate Professor at Duke University School of Nursing and Senior Fellow in the Center for the Study of Aging and Human Development.

**Reuben R. McDaniel, Jr.,** EdD, is the Charles and Elizabeth Prothro Regents Chair in Health Care Management, Department of Management Science & Information Systems in the McCombs School of Business at The University of Texas at Austin.

**Cathleen Colón-Emeric, MD, MHS**, is Associate Professor of Geriatrics at the Durham Veterans Affairs Geriatric Research Education and Clinical Center and Associate Professor of Geriatrics in the Duke University School of Medicine, Department of Medicine, Division of Geriatrics.

## Pre-publication history

The pre-publication history for this paper can be accessed here:

http://www.biomedcentral.com/1472-6963/14/244/prepub
